# Supplementary material for: Method-specific beliefs and subsequent contraceptive method choice: Results from a longitudinal study in urban and rural Kenya
Source: PLoS One. 2021 Jun 18;16(6):e0252977. doi: 10.1371/journal.pone.0252977 (PMC8213138; doi:10.1371/journal.pone.0252977)
Supplement: Attachment — Submitted filename: 5 - PONE-D-20-34145_ReviewerComments.docx [file pone.0252977.s001.docx]

**PLoS One Review**

**Title**: Method-specific beliefs and subsequent contraceptive method choice: results from a longitudinal study in urban and rural Kenya

**Manuscript Number**: PONE-D-20-34145

**OVERALL FEEDBACK**: This study examines an important and timely topic—the relationship between contraceptive beliefs and adoption of specific contraceptive methods. The authors explore a range of different characteristics specific to the three methods explored as outcomes (implants, injectables and pills), which builds substantially on a currently limited literature base. Clarification of the distinct study samples, derived from the cohort study, would improve the methods section; use of a study selection flowchart is recommended. As described in the feedback for the methods section, it is unclear why the authors used follow-up data from different survey rounds when comparing Nairobi and Homa Bay, thereby inducing systematically different follow-up times between the samples. Given the authors’ inclusion of women who were pregnant at enrollment (more than one-quarter of non-users at baseline in both samples), I expected to see some assessment of or adjustment for postpartum status, as this is likely to shape how women act on beliefs to make decisions about contraception. Other specific suggestions, which I hope will improve the clarity and contribution of this important work, are itemized by section. A few grammatical errors, verb tenses, and missing words are also present throughout the manuscript and require attention before finalization.

**ABSTRACT**

- On line 29, the authors use “round 2” to talk about follow-up data collected as part of the mentioned longitudinal study. This terminology is confusing because the study design has not yet been described. Please review the abstract accordingly.

**INTRODUCTION**

- On line 54, the authors state: “Fear of side effects, damage to health, menstrual disruption, and long-term infertility have been widely documented, including for Kenya [6-8].” Sentence should be clarified to state that these are the barriers mentioned in the previous sentence.

**MATERIALS AND METHODS**

- On line 94, please define acronym “NUHDSS” at first use.
- Lines 103-110 describe how the authors arrived at the analytic sample. A figure would be helpful here, particularly since a broader sample is included in Tables 1-2, compared to the final analytic sample for the specific contraceptive outcomes in Tables 3-5. This would be especially helpful given the difference in follow-up times between Nairobi and Homa Bay samples.
- On lines 103-106, the authors describe the cohort study samples from the two sites. While there was no “round 3” conducted in Nairobi, why did the authors choose to use the different follow-up rounds for the two sites? Why not just restrict them both to round 2, given that the follow-up time between the baseline and follow-up samples would be more comparable? In the methods description, it does seem like round 2 data is available in Homa Bay, so this should be used instead of round 3. If not adjusting for time since baseline belief in each model, both models should at least include comparable samples in terms of follow-up time.
- On line 112, the authors state, “…1866 women during the third round (91% of those interviewed in round 2).” This should be stated in terms of the retention from the original sample, not the round 2 sample. This recommendation can be ignored if the sample for Homa Bay is revised to include round 2 as follow-up only.
- On line 125-126, the authors state, “Note that beliefs b and c are valid while beliefs a, d,

and e are erroneous.” Why do the authors make the distinction between beliefs about contraception that are (in their mind) “valid” and others are considered “erroneous”? If these beliefs are shaped by women’s social networks, communities, etc. and, in turn, inform women’s contraceptive decision-making, then why is this distinction important? Additionally, the authors should include some mention of the large body of literature which ties contraceptive use/non-use to beliefs about contraception and infertility.

- On line 126, the authors mention, “Because of evidence on the importance of social influences…” Please cite relevant literature here or in the introduction.
- On lines 130-131, the authors mention that, “Women's perception of their partners' approval of the method was also ascertained; in the analysis, don't know responses were combined with disapproval.” Why were “don’t know” responses combined with disapproval and not approval? For what proportion of the sample did this affect? I am particularly interested in this given the significant findings discussed later in the manuscript, which show a strong influence of perceived partner disapproval and contraceptive choice in Nairobi. Was there a difference between the proportion of women who selected “don’t know” between urban and rural sites that may have related to the observed differences?
- On line 146, the word “variable” is meant to be plural (i.e., variables).
- Participants who were pregnant at baseline were included in the analysis, accounting for more than one-quarter of all non-users at baseline. Was there any assessment of if method choice was shaped by whether or not women were postpartum? I expected to see some assessment of or adjustment for postpartum status, as this is likely to shape how women act on beliefs to make decisions about contraception. I understand that sample sizes are certainly an issue, but feel strongly that postpartum status be recognized in this analysis.

**RESULTS**

- On line 178-179, the authors state: “In Nairobi, 74% were using a method of contraception at baseline compared with 65% in Homa Bay.” In the methods section, the analytic sample is described to be women who were *not using contraception* at baseline, given that the focus of the analysis is contraceptive method adoption between baseline and follow-up. It is confusing to have Tables 1-2 present data for the full sample of women from the existing cohort, as many of them are not eligible for participation in the present study since they are not “at risk” for the outcome.
  - If Tables 3-5 present results for the subsample of women who were not using at baseline, but had adopted an injectable, pill or implant by follow-up, Table 1 should reflect these same populations for comparison. For example, if the authors feel it is important to retain information on the full cohort in this paper, Table 1 might show three categories in each site: contraceptive users at baseline vs. non-users at baseline who do not adopt implants, injectables or pills vs. non-users at baseline who adopt implants, injectables or pills (i.e., analytic sample for Tables 3-5, main analyses). This would make it easier, from a reader’s perspective, to compare the population of women in the analytic sample to other women surveyed.
- With the previously suggested revision, update lines 176-186 to provide comparison between the analytic sample and the other two samples derived from the same cohort.
- In Table 3, please define “ns” in the notes under the table as “non-significant”.
- Tables 4-5 should include Ns to specify analytic sample sizes.
- Clarify throughout the results—and in other sections—when “husband’s approval” is stated that this is “perceived husband approval”, as this measure is reported by the woman herself (e.g., lines 240, 260, etc.)
- Line 276 includes a typo for the word “above”

**DISCUSSION**

- The paragraph articulated in lines 309-317, connection between prior contraceptive use and future use relating to unmet need and programs, is excellent. Bravo!
- Starting on line 329, the authors focus the discussion on the contrast observed in the relationship between contraceptive beliefs and method-specific use across the two sites. See earlier comment in methods section about making these two samples more comparable with the use of round 2 data from Homa Bay to reflect the same follow-up times between baseline and follow-up for the two samples.
- Starting on line 336, the authors describe the perplexing association between perceived husband’s approval and method choice, comparing the two sites. See earlier comment in methods section about the proportion of women who responded “don’t know” and how they were reclassified as having partners that “disapproved” of the method. Given these inconsistent results across the two sites, I remain curious about the proportion of women in each site whose responses were reclassified and how this reclassification might have misclassified women with uncertainty about their partners’ beliefs as having
  “disapproving” or hostile partners and therefore shaped the observed results.
- On lines 344-346, the authors note that, “Beliefs about menstrual disturbance and safety for long-term use were related to method choice in the rural sample but not in Nairobi”. Perhaps women in Homa Bay value menstrual regularities more than women in Nairobi, seeing menstrual disturbances as a reflection of poor health, potential challenge in their relationships, potential indicator of later infertility, etc. whereas women in Nairobi conceptualize menstrual disturbances differently (e.g., that no bleeding is good, not a sign of poor health). See Polis 2018, ‘“There might be blood”: Scoping review of women’s responses to contraceptive-induced menstrual bleeding changes’ and other articles focused on how changes in menstruation affect women differently.
- On lines 346-348, the authors highlight how the sites are distinct, indicating that their comparison might not be appropriate. Given the focus on the site-specific differences throughout the results and flagged in the abstract, this statement should come earlier in the article. Authors should clearly indicate that the two sites were selected due to the availability of data in the cohort study, etc. and not due to their comparability overall.
- I appreciate the authors’ focus on the negative results in lines 349-361. This is an important contribution—equally as valuable as the positive results, as the authors note!
- As flagged in the methods, the authors should reference literature on social networks and contraception to contextualize the results outlined in lined 358-361.
  - On line 367-368, the authors state that women’s contraceptive beliefs may have changed “perhaps because of an unsatisfactory experience of a social network member”, which affected the insignificant relationships between many beliefs and later use of methods. Given the last point about the lack of observed association between social network experiences and women’s contraceptive choices, I don’t think this makes sense as a plausible explanation.

**CONCLUSION**

- On lines 383-384, the authors end with “Women's choices about which method to use should be based, as far as possible, on correct knowledge.” This seems like a bit of a leap. The focus should not be on women’s “correct knowledge” but instead on how valid concerns, misconceptions, fears, etc. of contraceptive attributes require attention from the field.
